# Pulmonary Embolism Originating from a Hepatic Hydatid Cyst Ruptured into the Inferior Vena Cava: CT and MRI Findings

**DOI:** 10.1155/2016/3589812

**Published:** 2016-01-21

**Authors:** Necdet Poyraz, Soner Demirbaş, Celalettin Korkmaz, Kürşat Uzun

**Affiliations:** ^1^Department of Radiology, Meram Faculty of Medicine, Necmettin Erbakan University, Konya 42080, Turkey; ^2^Department of Pulmonary Disease, Meram Faculty of Medicine, Necmettin Erbakan University, Konya 42080, Turkey

## Abstract

Pulmonary embolism due to hydatid cysts is a very rare clinical entity. Hydatid pulmonary embolism can be distinguished from other causes of pulmonary embolism with contrast-enhanced computed tomography (CECT) and magnetic resonance imaging (MRI). MRI especially displays the cystic nature of lesions better than CECT. Here we report a 45-year-old male patient with the pulmonary embolism due to ruptured hydatid liver cyst into the inferior vena cava.

## 1. Introduction

Cystic echinococcosis (CE), or cystic hydatidosis, is a complex, chronic parasitic disease with a cosmopolitan distribution. Human CE remains highly endemic in pastoral communities,particularly in regions of South America, the Mediterranean littoral, Eastern Europe, the Near and Middle East, East Africa, Central Asia, China, and Russia [1–3]. Cystic echinococcosis is caused by the larval stage (metacestode) of* Echinococcus granulosus* in sheep raising areas with close contact to dogs. Organisms that reach the gastrointestinal system go to the liver via the portal vein and then to the right heart and to the lung via the pulmonary artery and may reach the spleen, muscles, central nervous system, or eye via systemic circulation [1, 2, 4]. In humans, 75% of HCs are seen in the liver, 15% in the lungs, and 10% in other anatomical locations. The cardiovascular system may also be involved in less than 2% of the cases [4].

We report a case of a 45-year-old man with hydatid cyst embolization to the pulmonary arteries mimicking the clinical presentation of acute pulmonary embolism.

## 2. Case Presentation

**Figure 1:**
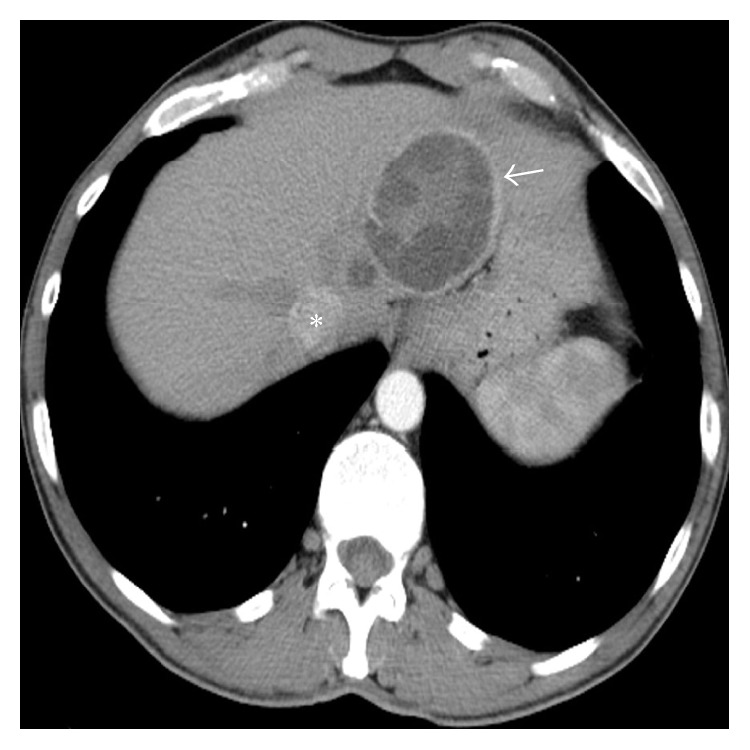
Contrast-enhanced CT scan reveals typically hydatid liver cyst as CE 3a cyst following the WHO classification (arrow) adjacent to the IVC (asterisk).

**Figure 2:**
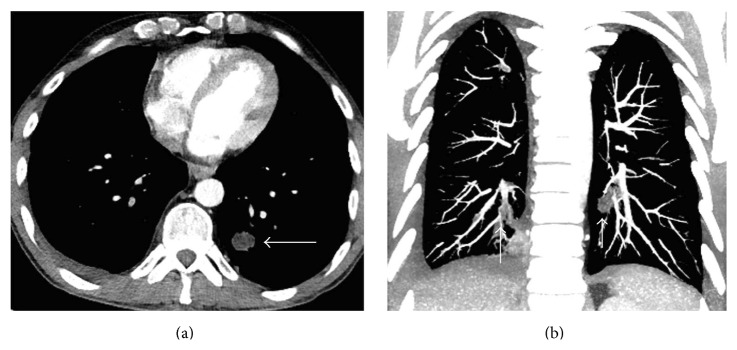
Axial contrast-enhanced CT scan (a) and coronal MIP image (b) show multiple cystic nodules in the inferior pulmonary arteries (arrows).

**Figure 3:**
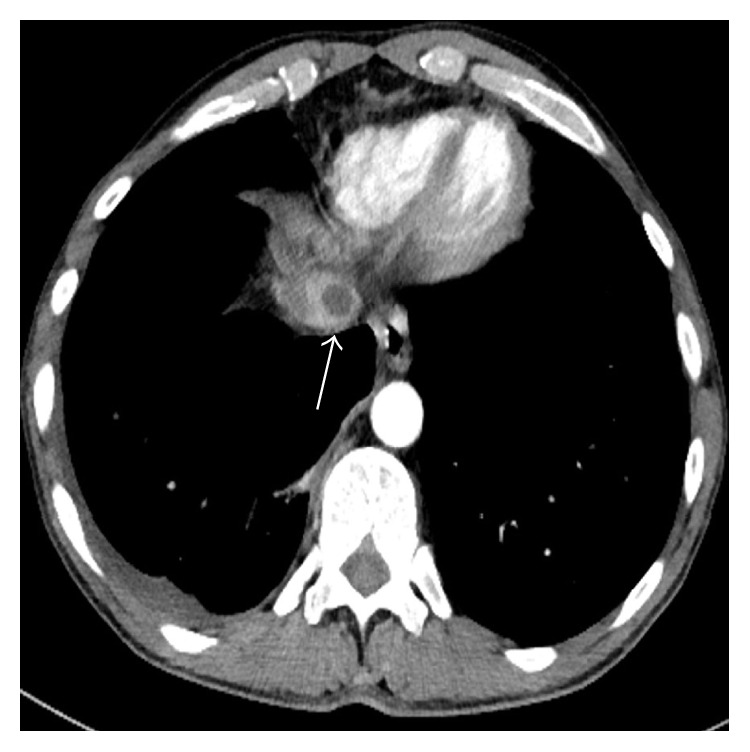
Contrast-enhanced CT scan shows a fluid attenuation round lesion, daughter cyst, within the IVC (arrow).

**Figure 4:**
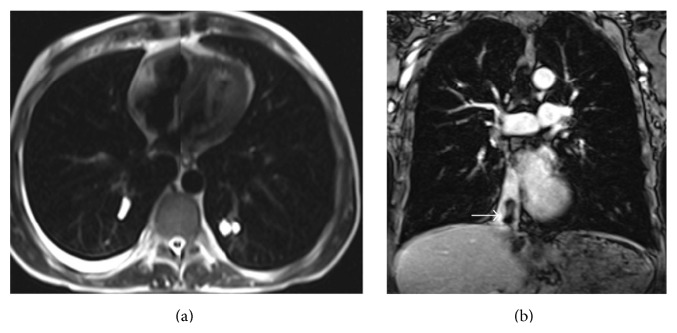
Axial T2-weighted MRI shows hyperintense cystic nodules in the lower lobe segmental pulmonary arteries bilaterally (a). Contrast-enhanced T1-weighted Vibe sequence coronal MRI shows contrast filling defect within the IVC due to ruptured liver hydatid cyst (b) (arrow).

A 45-year-old male patient was referred to our clinic with symptoms of chest pain and dyspnea at rest. There was no history of cardiovascular diseases. Physical examination was unremarkable. Electrocardiogram traces demonstrated a sinus tachycardia. The D-dimer test was normal. From past medical history, he had cystic echinococcosis in the liver two years ago, but the patient refused surgical intervention and for this reason he was given medical treatment with albendazole alone. Since then he did not have check-up. A pulmonary embolism was clinically suspected. Chest radiography showed several nodules in the lower lung zones. The CECT revealed a typical HC with the size 63 × 53 mm, containing peripherally aligned small cysts, adjacent to the inferior vena cava in the liver as CE 3a cyst following the WHO classification (Figure 1). In the lower lobe segmental branches of the pulmonary arteries bilaterally, multiple cystic nodules that caused luminal widening consistent with emboli were observed (Figure 2). A cyst that caused a filling defect was noted in the lumen of the inferior vena cava (Figure 3). Pleural effusion was present in the right hemithorax. The patient did not have any predisposing factors of thromboembolic diseases or any findings of deep vein thrombosis in the lower extremities. The thoracic MRI performed to confirm the findings revealed septation in the liver and HC containing multiple daughter vesicles aligned peripherally, and the cystic structure of the emboli inside the inferior vena cava and pulmonary arteries was shown on MR images (Figure 4).

## 3. Discussion 

Pulmonary or systemic embolisms caused by HCs are rare complications. Hepatic echinococci may open to the inferior vena cava, and daughter vesicles may cause embolisms in the pulmonary arteries. Sometimes cardiac HC can rupture directly into the pulmonary arteries [4, 5]. In these cases, a clinical presentation similar to acute pulmonary thromboembolism with coughing, hemoptysis, and chest pain develops [5]. These cysts might mechanically block blood flow. When supportive blood nutrition is provided by bronchial arteries, the pulmonary artery obstruction caused by slow-growing cysts may remain asymptomatic. Finally, the progression of the disease may cause symptoms like dyspnea, hemoptysis, and chest pain, and anaphylactic shock may develop due to leakage of the hydatid cyst fluid. Early diagnosis with imaging studies and treatment are the main aspects of preventing complications [6–8].

The diagnosis of HC pulmonary embolism can be made with clinical and radiological findings. On CECT, cysts causing focal widening of the lumen of arteries and not enhancing with contrast appear homogenous and hypodense at fluid density. MRI displays the cystic nature of lesions better than CECT [9]. The presence of HC inside the heart or liver (as in our case) makes the diagnosis easier. In our case, CECT and MRI successfully revealed the lower lobe pulmonary artery segmental branches bilaterally and the daughter vesicles of the cystic embolisms inside the inferior vena cava [6, 10].

Other reasons of intraluminal filling defects such as pulmonary thromboembolism and primary arterial tumors should also be considered in the differential diagnosis. Primary arterial tumors are more aggressive and enhance with contrast [7, 8].

In limited cases of pulmonary embolism caused by HCs surgery is recommended [6]. Our patient refused surgery; therefore, he was discharged on oral albendazole therapy and is still being followed up.

In conclusion, incorrect and unnecessary treatments can be avoided by distinguishing pulmonary emboli secondary to HCs from pulmonary thromboembolisms.
